# Financial performance evaluation by multi-criteria decision-making techniques

**DOI:** 10.1016/j.heliyon.2022.e09361

**Published:** 2022-05-03

**Authors:** Nida Türegün

**Affiliations:** Ozyegin University, School of Applied Sciences, Turkey

**Keywords:** BIST, Tourism sector, Financial performance, Entropy, TOPSIS, VIKOR

## Abstract

A thorough review of techniques for the experts invested in capital markets is necessary to take the decision-making process on the stock. When it comes to profiting from the capital markets, timing is crucial. The accurate evaluation of the financial performance of the businesses in the tourism sector is of great importance both in socio-economic and strategic terms in all countries in the world. As a result, the majority of investors use multi-criteria decision-making techniques to choose the best stocks. Thus, this paper aims to perform analysis on the TOPSIS, and VIKOR multi-criteria decision-making methods by taking base as an entropy method across companies that operate in the tourism industry and are publicly traded on the Borsa Istanbul by covering the data from 2018 to 2020, and to uncover the performance results of the companies and rank them by these main criteria. In the analysis results regarding the evaluation of the financial performance of tourism companies traded in BIST, it was seen that the ranking results made with TOPSIS and VIKOR methods were similar in 2018 and 2019. It is slightly different in 2020. It was seen that AVTUR was the most important alternative in both methods, whereas MARTI had the lowest ranking alternative. Moreover, MERIT, KSTUR, and PKENT have been determined as fluctuating companies.

## 1 Introduction

The majority's stock selection decision is often reflected in stock charts, and investors prefer to be buyers or sellers, depending on demand patterns and stocks with strong trends. Professionals, on the other hand, make the bulk of their investment decisions based on economic and firm-based metrics, as well as historical stock data. Accurate assessment of companies in the industry may represent the position of various firms as they compete with each other, specifying benefits and drawbacks, prospects and challenges for firms (Carmona et al., 2014). Firm assessment is an important industrial function. Investors are constantly searching for the right investment field for the benefit of further interest. As a result, they are constantly attempting to analyze and differentiate between successful and unsuccessful firms. Timing is an essential aspect of investing to maximize the benefits from the financial markets. The critical aspect of the profitable decision-making strategy is the ability to make the same decision alongside or ahead of the rest of the market.

Multi-criteria decision-making methods (MCDM) are one of the most widely used industry trend predictors among academics and investors. MCDM is a technique that combines the output of alternatives with a variety of conflicting, qualitative, or quantitative parameters, resulting in a consensus-based solution (Zaremba, 2019). Knowledge from a variety of disciplines is included (e.g. behavioral decision, computational technology, economics, information management, and mathematics). Many MCDM techniques and methods have been successfully developed, suggested, and implemented in several application areas since the 1960s. The aim of MCDM is not to propose the right decision, but rather to help decision-makers to choose selected alternatives or a single source that meets the criteria of their choices. It has been noted that, for efficient and successful decision making, it is essential to know about the MCDM approaches and to have sufficient information about the viewpoints of the players engaged in decision-making processes (Chan et al., 2014). Many scholars used MCDM in diverse fields in decision-making during the last decade. Each of the methods is similarly qualified for making decisions in an unpredictable environment, and each method has its own set of benefits.

For nearly three decades, tourism has been the most rapidly expanding industry in Turkey. A reliable and effective financial performance evaluation plays an important role for a company that aims to retain its marketplace and defend its market shares against any future challenges in today's highly competitive climate. The most important feature that distinguishes the tourism industry from other industries is its highly leveraged ability to create employment quickly (Yabuuchi, 2013). The Turkish economy has become increasingly important among the ranks of the global economic actors on a regular basis throughout the past few decades, as it can generate faster-growing employment opportunities compared to similar emerging countries and regions. The tourism sector continues to be a highly invested and developing sector in the world economy (Chung and Parker, 2010). The progress of the tourism sector has been undeniable in recent years. Understanding the level and the fundamental variables of this progress is very important to shed light on the contribution of the sustainable competitive advantage of the tourism sector not only to the industry itself but to the national economy as well.

Tourism companies that conduct business in a competitive marketplace also use financial forecasting and analysis of their market positions in order to boost their potential financial results. As said by Halkos and Salamouris (2004), conventional ratio analysis is not adequate to calculate the financial results of businesses and can use multi-lateral approaches. As a multi-lateral approach, this paper aims to perform analysis on the TOPSIS, and VIKOR multi-criteria decision-making methods by taking base as an entropy method across companies that operate in the tourism industry and are publicly traded on the Borsa Istanbul (BIST) by covering the data from 2018 to 2020, and to uncover the performance results of the companies and rank them in the tourism sector by these main criteria. It is important to determine the attribute weight according to the TOPSIS and VIKOR method procedures. There are several ways for determining weight, including linear programming techniques for multidimensional analysis of preference (Srinivasan and Shocker, 1973), weighted least square method (Chu et al., 1979), analytic hierarchy process (Saaty, 1980), entropy method (Hwang and Yoon, 1981), criteria importance through inter-criteria correlation (Diakoulaki et al., 1995), and rank correlation analysis method (Guo, 2012). In the TOPSIS and VIKOR methods, the entropy approach is typically employed to determine the attribute weight (Shemshadi et al., 2011; Liu et al., 2018; Roy and Das, 2018; Narayanamoorthy et al., 2019; Chen, 2019; Chai et al., 2019; Yildiz, 2020). The term ‘entropy-based TOPSIS and VIKOR’ is used in this study. The entropy-based TOPSIS and VIKOR method is an objective approach in which the weighting and decision results are determined only based on objective data of alternatives. In this study, the analysis is focused on this approach.

Besides, this study attempts to link the gap in the literature by presenting the similarities and comparisons between entropy-based TOPSIS and VIKOR methods to analyze the performance of the tourism companies, which is a study area that was previously unexplored. Moreover, the utilization of growth and market rates and their choice of weighting in the study are distinguishing factors that set apart this study from previous work in the body of research. This study comes forth as an example and provides direction for the methods that can be applied not only in the tourism sector but also in other sectors. Furthermore, this study provides significant support to investors in making the right decisions by measuring the ranking of companies in the sector with two different techniques.

The rest of the paper is organized as follows. Section 2 presents the motivation and contribution of the study. Section 3 gives a brief explanation of the background of TOPSIS, VIKOR, and entropy methods and related works for the methods. Section 4 displays the performance analysis according to the steps of the analysis. Section 5 gives the results and discussion and section 6 presents the conclusion.

## 2 Motivation and contribution

Tourism has grown steadily and geographically diversified over the last decades, making it one of the fastest-growing and largest economic sectors in the world, contributing almost 10 percent to the world's gross domestic product and creating one in eleven global jobs. The strong interconnection between tourism and other economic activities, comparatively low entry costs, and future local benefits are expressed in the fact that tourism seems to be the only service sector. Developing countries have great importance in this growing sector. Currently, 45% of all international tourist arrivals are accounted for by emerging and developing economies and this proportion is projected to hit 57% by 2030. In those countries, the tourism sector contributes to economic growth and provides considerable additional opportunities. Tourism is employment concentrated and has connections with numerous other areas of the economy. It directly contributes to the reduction of poverty, especially among women, acknowledged both at the national and international level by policymakers (ITC/UNWTO, 2015).

As an instrument for growth and a catalyst for socio-economic change, tourism is known for having various features that make it extremely valuable. Tourism creates ripple impacts through many other business operations in the tourism supply chain, penetrating the local economy and extending the productivity influence of exports since it spans a wide variety of goods and services industries. Tourism was recognized as the main sector for development in the Istanbul LDC Programme of Action (Diallo et al., 2020). Tourism touches on a wide variety of diverse aspects of strategy. The spending of foreign tourists is counted as exports to the destination country and as imports to the visitor's country of residence. The role of the tourism industry is gradually gaining prominence both in business and in growth among the world's decision-makers.

The tourism industry creates economic entities that try to fulfill the demands by creating tourism services and to get the ultimate profit. In order for tourist enterprises to thrive, continue their operations and attain their goals, it is vitally significant to use information that is gathered when reviewing, assessing, and establishing future plans for financial performance. The financial performance evaluation involves investors and creditors. Financial performance evaluation also gives crucial information to decision-makers, such as managers, who use it to assess the past and set goals for the future. In addition, BIST is a growing stock exchange, which takes the investors’ interest. The BIST 100 increased 83 points or 5.60% since the beginning of 2021 (Trading Economics, 2021) There are many analyzes made for other sectors of the BIST, however, there are few analyzes made for the tourism sector. The fact that the subject of this study has not been researched previously in the tourism sector of the BIST was the driving force and motivation behind it.

This study has 3 contributions. First, it contributes tourism sector and literature by analyzing companies with multi-criteria decision-making methods. It will serve the national and international spotlight that provides a unique opportunity for investors to identify tourism companies’ performances to make better strategic decisions. Strategic decision-making requires measurement. Thus, this study displays statistical information on tourism companies which is essential in advancing knowledge of the sector. Besides, considering MCDM approaches are used as supplementary instruments by decision-makers, it is more suitable to use them to assess all approaches rather than to recommend a single method. The second contribution of this study is that multiple ranking alternatives within the same level can be generated using the same data, and these ranking alternatives will allow decision-makers to make alternative evaluations. The third contribution is to provide accurate information to investors by displaying analysis results of the similar financial performances of the tourism companies that are listed on the BIST over the years. Entropy-based TOPSIS and VIKOR methods have contributed to performance measurement by bringing together many variables. In addition, the use of growth rates and market rates used in determining the weights in the study is a different factor that distinguishes this study from other studies. If we give examples of studies that take this study into account using multi-criteria decision-making methods, many sectors such as the automotive industry and the food sector can be analyzed.

## 3 Background and related work

In this section brief explanation of the background of TOPSIS, VIKOR, and entropy methods and related work is presented in order to better understand the general framework of the study.

### 3.1 TOPSIS

The use of mathematical techniques, seen as the basis of MCDM techniques, dates back to the 1700s. These techniques are mentioned in the works of researchers such as Benjamin Franklin, Marquis de Concordet, Francis Edgeworth, and Vilfiredo Paredo. In the last century, developments in the field have risen above the work of many economists, mathematicians, and scientists from other disciplines such as Frank P. Ramsey, Leonard Savage, Jon von Neuman, Oscar Morgenstern, John Nash, Paul Samuelson, Ward Edwards, Herbert A. Simon (Koksalan et al., 2011). MCDM methods, which are common for the purpose of "optimization" in mathematical techniques developed by inspiring from the solutions of the problems of the distribution of military resources in the most appropriate way, have shed light on the solution of many social, financial, and economic problems.

TOPSIS (Technique for Order Performance by Similarity to Ideal Solution) method is one of the well-known multi-criteria decision-making methods developed by C.L. Hwang and K. Yoon in 1981 (Hwang and Yoon, 1981). Yoon in 1987, and Hwang et al., in 1993 contributed to the development of the concept (Yoon, 1987; Hwang et al., 1993). TOPSIS appeals to decision-makers since it requires less subjective input from them. Only weights are required for subjective input. TOPSIS is a valuable approach for solving real-world multi-attribute or multi-criteria decision-making issues. It assists decision-makers in organizing issues to be solved as well as conducting analyses, comparisons, and rankings of options. Likewise, it's a balancing strategy approach that analyzes a group of alternatives by determining weights and normalizing scores for each criterion, and computing the mathematical distance among each and the ideal alternative, which has the highest score in each criterion (Yoon and Hwang, 1995). TOPSIS makes the premise whether the criteria are increasing or decreasing monotonically. In multi-criteria situations, normalization is generally essential since the parameters or criteria are often of conflicting dimensions. TOPSIS and other compensatory approaches allow for trade-offs between criteria, where a bad outcome in one criterion might be offset by a good result in another. Non-compensatory approaches, which include or exclude alternate solutions based on hard cut-offs, give a more realistic type of modeling.

The basis of the method is to choose the alternative that is the shortest distance from the most suitable (ideal) solution point and the furthest from the worst (negative ideal) solution point (Yoon and Hwang, 1995). It is one of the widely used methods because of its rational and understandable logic, limited subjective input, and many features such as determining the best alternative in the fastest way and adding the relative weights of criterion importance (Ulas and Keskin, 2017). The TOPSIS method became popular and successful among the different MCDM methods due to its basic computational steps, its strong quantitative bases, and its method which is simple to understand (Yildiz, 2020). This process classifies the alternatives according to the distance between the ideal positive and negative solutions. Nevertheless, a selected alternative is supposed to be away from the negative ideal solution and close to the ideal solution. The TOPSIS method's optimal alternative is the one that comes nearest to the positive ideal solution. The system of TOPSIS can be used for assessing decision-making bodies (Ghosh and Saima, 2021).

In this study, the TOPSIS procedure is carried out in 6 steps. According to the procedure, step 1 creates a decision matrix, step 2 performs the normalization process, step 3 constructs the weighted normalized matrix, step 4 creates ideal and negative ideal solution points, step 5 calculates distances to the maximum ideal point and step 6 computes the relative proximity to the ideal solution. The steps and their detailed explanations are shown in Table 1 in section 4 (see Table 2).

**Table 1:** Steps of the methods.

		Formula	Explanation
**ENTROPY**			
Step 1	Creates a decision matrix		A decision matrix is created for a multi-criteria decision problem with m alternatives and n criteria. Where: X_ij_: i is alternative, j is the success value according to the criteria, i = 1,2… m and j = 1,2…, n (Equation 1)
Step 2	Performs normalization process		At this step, since the criteria have different scales, the normalization process is carried out first, and this is done using the following equation. Here; *i* = alternatives, *j* = criteria, r_*ij*_ = normalized values, x_*ij*_ = utility values. (Equation 2)
Step 3	Entropy values for criteria are found.		the entropy values of the determined criteria are calculated at this step. (Equation 3)
Step 4	The degree of differentiation (dj) of information is calculated		Here the value of k is a constant defined by k = 1/lnm and guarantees condition 0 ≤ e_j_≤1. By using the entropy value, the degree of differentiation d_j_, its values are calculated for each criterion as in the formula. (Equation 4)
Step 5	The entropy weights of the criteria are calculated.		At this step, the objective weights of each criterion are calculated using the following equation. (Equation 5)
**TOPSIS**			
Step 1	Creates a decision matrix		The decision matrix created by the decision maker is a matrix of size m x n. i is success values of alternative according to all criteria, column x_jj_ are the success values of all alternatives according to the criteria. (Equation 6)
Step 2	Performs normalization process		At this step, it is ensured that the matrix is normalized by taking the square root of the sum of squares of the values of the criteria in the decision matrix. An element of the normalized decision matrix is denoted by "*r*_*ij*_". (Equation 7)
Step 3	Constructing the weighted normalized matrix		Multiply the previously obtained normalized matrix with the criterion weights matrix that has been previously determined or calculated by another technique. (Equation 8)
Step 4	Creation of ideal (A ∗) and negative ideal (A⁻) solution points		Here, the maximum and minimum values in each column in the weighted matrix are determined. (Equation 9 and Equation 10)
Step 5	Calculation of distances to the maximum ideal point		Euclidean metric is used for distance calculation. (Equation 11 and 12)
Step 6	Computing the relative proximity to the ideal solution		The relative proximity of each decision point to the ideal solution is calculated and indicated by. The alternatives are sorted by ranking the C_i_∗ values from the highest to the lowest. (Equation 13)
**VIKOR**			
Step 1	Creating the decision matrix		Decision matrix is created such that rows show alternatives (m) and columns show criteria (n). (Equation 14)
Step 2	Detection of the best and worst values		The best (f∗) and worst (f^−^) values are determined for each criterion. If j. If the criterion has the utility property, the parent formula, if j. If the criterion has the cost property, a sub formula is used. (Equation 15, 16, 17 and 18)
Step 3	Creating the normalized decision matrix		Linear normalization process is applied in this step. (Equation 19)
Step 4	Creating a weighted decision matrix		The weighted normalized matrix is obtained by performing the same operations with the 3^rd^ step of the TOPSIS method. (Equation 20)
Step 5	Calculation of Sj and Rj values of each alternative		*i* denotes mean and worst group scores for alternative (Equation 21 and 22)
Step 6	Calculation of Qj values for each alternative		Here, parameter q is the weight of the majority of the criteria, that is, the weight for the strategy that provides the maximum group benefit, and the parameter (1-q) is the weight of the minimum regret. Compromise is achieved by q˃0.5 majority vote, q = 0.5 consensus, or q˂0.5 veto. (Equation 23)
Step 7	Ranking and auditing alternatives		By listing the values of S_i_, R_i_, Qi, three separate lists are obtained and then the accuracy of the ordering is tested. For the test, it is checked whether the alternative with the Q_i_ value satisfies the two conditions. Condition 1: Acceptable advantage. Qi is ordered in ascending order of values and acceptable condition for the first two alternatives A1 and A2 DQ = 1/m-1 (number of alternatives in m) Condition 2: Acceptable Stability Condition: when the values are sorted from small to large, the first It is the alternative that takes the minimum value in the ordering made according to alternative S and/or R values. The best alternative in the ranking based on Q values is the alternative with a minimum Q value. (Equation 24)

**Table 2:** Ratio groups.

Group	Abbreviation	Name	Formula
Liquidity Ratios	CR	Current Ratio	Current Assets/Current Liabilities
	AR	Acid-Test Ratio	Current Assets-Inventory/Current Liabilities
	CAR	Cash Ratio	Liquid Assets/Current Liabilities
Activity Ratios	AT	Asset Turnover	Net Sales/Total Assets
	CT	Capital Turnover	Net Sales/Equity
	ART	Accounts Receivable Turnover	Net Sales/Trade Receivables
	IT	Inventory Turnover	Cost of Goods Sold/Inventory
Solvency Ratios	LR	Leverage Ratio	Total Liabilities/Total Assets
	DER	Debt to Equity	Total Liabilities/Equity
	LAR	Current Liabilities/Total Assets	Current Liabilities/Total Assets
Market Ratios	PBR	Price to Book Ratio	Market Value/Book Value
	PSR	Price to Sales Ratio	Price/Sales
	PER	Price to Earnings Ratio	Price/Earnings
Profitability Ratios	RA	Return on Assets	Net Income/Total Assets
	RE	Return on Equity	Net Income/Equity
	GPM	Gross Profit Margin Ratio	Gross Profit Margin/Net Sales
	NPM	Net Profit Margin	Net Income/Net Sales
Growth Rate	AGR	Asset Growth Rate	(Total Assets_t_-Total Assets_t-1_)/Total Assets_t-1_
	EGR	Equity Growth Rate	(Equity_t_-Equity_t-1_)/Equityt-1
	SGR	Sales Growth Rate	(Sales_t_-Sales_t-1_)/Sales_t-1_

### 3.2 VIKOR

The VIKOR method is a multi-criterion decision-making approach. VIKOR positions alternatives and chooses the compromise solution that is closest to the ideal. Serafim Opricovic has initially developed the method. His motivation was to solve decision problems with contradictory criteria. He assumed that compromise is meaningful for conflict management. The decision-maker seeks a solution that is as close to ideal as possible, and the options are weighed against all stated criteria. Po-Lung Yu and Milan Zeleny proposed the concept of compromise solution in multi-criterion decision-making in 1973 (Yu, 1973; Zeleny, 1973). In his Ph.D. dissertation in 1979, S. Opricovic devised the core principles of VIKOR, and implementation was presented in 1980 (Duckstein and Opricovic, 1980). In 1990, the word VIKOR was coined from the Serbian phrase Vise Kriterijumsa Optimizacija I Kompromisno Resenje, which translates to "Multicriteria Optimization and Compromise Solution" and is pronounced "VIKOR" (Opricovic, 1990). In 1998, the actual implementations were demonstrated (Opricovic, 1998). The publication published in 2004 contributed to the VIKOR method's international recognition (Opricovic and Tzeng, 2004).

The VIKOR method was created to optimize complex systems using multiple criteria. It specifies the compromise order list, solution, and the weight stability range for the chosen stability in the achieved compromise solution with the original weights. This method concentrates on ordering and choosing several alternative solutions when there are multiple criteria to consider. It provides the 'ideal' solution with a multi-criteria rating index based on the 'closeness' metric (Opricovic, 2015). This method is a useful device in MCDM, particularly where decisions-makers at the outset of system design cannot express their preferences. This method is based simultaneously on ordering and choosing from a variety of alternatives. It specifies a compromise solution for contradicting criteria issues that can assist decision makers in reaching a very last solution. VIKOR is useful when the system design is not known by the decision-maker. One of the most important advantages of the method is that it does not need to add predetermined views to evaluate options based on existing criteria and can use raw data directly. The decision-maker accepts the compromise solution achieved, which benefits the majority as a group and minimizes the opponents' regrets (Pekkaya, 2015).

In this study, the VIKOR procedure is carried out in 7 steps. According to the procedure, step 1 creates a decision matrix, step 2 detects the best and worst values, step 3 creates the normalized decision matrix, step 4 creates a weighted decision matrix, step 5 calculates Sj and Rj values for each alternative, step 6 calculates Qj values for each alternative, and step 7 ranks and audits alternatives. The steps and their detailed explanations are shown in Table 1 in section 4.

### 3.3 Entropy

The entropy method is a standard weighing approach based on diversity, which determines the weights of the attributes based upon the variety of data between the alternatives (Chai et al., 2019). The concept of entropy was first introduced by Rudolp Clausius in 1865. The idea of entropy arose in response to the fact that a specific quantity of functional energy generated by combustion processes is constantly lost due to dissipation and so is not converted into productive work. He proposed the thermodynamic system and argued that in any irreversible procedure, a little quantity of heat energy is steadily wasted across the system border. Clausius expanded on his thoughts about wasted energy and developed the word entropy. The first two laws of Thermodynamics were introduced by Clausius. The first law states that the universe's total energy is constant, and the second law states that the universe's total Entropy is growing into a maximum value (Clausius, 1865).

In 1947, the concept is proposed in the work of Shannon and Weaver (1947). It has been developed as a neutral method for weight allocation based on the decision matrix without affecting the decision-making choice (Ahn et al., 2019). According to Shannon, entropy is a concept that refers to the degree of disorder in a given source. The greater the degree of disorder, the bigger the source's information potential (Shannon 1948). Furthermore, the concept was emphasized by Zeleny in 1982 for determining the objective weights of qualities (Zeleny, 1982). According to Zeleny, entropy is a measure of information uncertainty derived from probability theory. It implies that a widespread distribution has greater uncertainty than one with a high peak. Besides, information entropy is a suitable statistic to utilize by the decision-maker when deciding between numerous possibilities with the same probability (Zeleny, 1982).

Weight determination is an essential step when utilizing TOPSIS and VIKOR methods. The entropy method is chosen for this study since it is straightforward in computation compared with the subjective weighting approach provided by AHP and does not need to take subjective preference into account. To compute weight, it simply needs objective data. To calculate Entropy, one must consider the weight of the significance of the attribute (λi), which is directly related to the amount of intrinsic information generated by a set of possible alternatives for each i-*ith* attribute, and in parallel to the subjectivity associated with the importance, the culture, psychology, and the environment in which the decision-maker lives (Zeleny, 1982). In this study, the entropy procedure is carried out in 5 steps. According to the procedure, step 1 creates a decision matrix, step 2 performs the normalization process, step 3 finds entropy values for criteria, step 4 calculates the degree of differentiation of information, step 5 calculates the entropy weights of the criteria. The steps and their detailed explanations are shown in Table 1 in section 4.

### 3.4 Related work

TOPSIS and VIKOR methods have a wide variety of real-world applications across different fields. In this section, studies on these methods are given.

The TOPSIS approach can be extended and commonly used in use for various assessment parameters. The TOPSIS approach is applied in numerous fields like automotive, textile, health, banking, and education sectors. The example works for these sectors are as follows. Jati (2012) displayed a ranking of Webometrics for universities worldwide. The author used two multicriteria decision analyses which are TOPSIS and VIKOR methods for ranking. Barrios et al. (2016), used the MCDM method to choose the best suitable medical equipment. The author used a hybrid model of AHP and TOPSIS to select the best-suited tomography equipment. Hatami-Marbini and Kangi (2017), used the TOPSIS method to rank seven automotive firms for investment purposes by using Tehran Stock Exchange data. Chitnis and Vaidya (2018), displayed a ranking of efficiency in the banking sector by using the TOPSIS method. Bathrinath et al. (2020), presented a hybrid MCDM technique similar to AHP-TOPSIS for identifying and analyzing potential hazards that cause accidents and important alternatives in the textile sector.

Some of the selected works from accounting journals are as follows. Ho (2004) employed the TOPSIS method for ranking Australia's major banks according to their performance. Keskin and Ulas (2017) examined the effects of privatization and deregulation measures on the efficiency of the 20 busiest European airports between 2010-14 by utilizing the multi-criteria method of TOPSIS and data envelopment analysis. Ulas and Keskin (2017) selected 20 countries for the 2010-14 period to compare their economic performances according to their growth rate. AHP is used as a weight measurement and the TOPSIS method was employed to rank the countries. Zaremba (2019) addressed the necessity to understand the distinctive nature of the business in building bankruptcy projection models. The author determined several ratios and analyzed them, and the AirRank methodology was developed utilizing the TOPSIS method. Yildiz (2020) used entropy as a weight measure and the TOPSIS method as a ranking method to rank the performances of two participation indices according to Islamic principles and six conventional indices based on their risks and returns between the 2015–2017 period. Ghosh and Saima (2021) analyzed and predicted Bangladeshi commercial banks' financial sustainability and resilience in response to the unfavorable impacts of the COVID-19 epidemic. They used 18 publicly traded banks in Dhaka Stock Exchange. TOPSIS and HELLWIG methods are used for ranking.

Meanwhile, the VIKOR approach is applied in decision-making analyses in numerous fields like automotive, textile, health, banking, and education sector, etc. The example of works for these sectors are as follows. Liu et al. (2013), suggest that the evaluation of health care waste disposal alternatives can be regarded as a complex MCDM issue that calls for multiple alternative solutions. They applied the VIKOR method for evaluating health care waste disposal methods. Mančev (2013), used the VIKOR method to examine the quality of the services offered by the libraries of the University of Nis. Wu et al. (2018) displayed an evaluation of the universal production efficiency of 16 main China's commercial banks, and rank them for the period 2007–2014, in a cross-efficiency interval considered by all weighting systems together with a comprehensive VIKOR model. Ortíz-Barrios et al. (2018) applied dispatching algorithms based on FAHP and VIKOR operational selection while taking account of set-up delays and transmission batches in the textile industry. Dang (2019), employed multi-criteria approaches of decision-making to assess the environmental quality of the nations of the OECD. The weight of criterion is determined by the entropy weight approach, and the VIKOR method is used to rate various OECD nations due to their environmental quality. Dev et al. (2020), used the entropy-based VIKOR method to select the preeminent composite between the materials. ENTROPY was used to measure criteria weights, and the method of VIKOR was used to rank the composites manufactured.

Some of the selected works from accounting journals are as follows. Ercan and Onder (2016) ranked the financial performances of five listed insurance firms in BIST between the 2010–2015 period by using the VIKOR method. Erdoğan et al. (2016) ranked the financial performances of food firms listed in BIST between the 2011–2014 period by using TOPSIS, VIKOR, and ELECTRE methods. Izadi and Taghva (2017) employed AHP and fuzzy VIKOR techniques to select a dynamic enterprise resource planning system. Ahmadvand and Tamalloki (2017) assessed and prioritized sharia conforming alternatives for shorter sales, and the best appropriate methodology to their implementation on the Iranian stock market is introduced through the VIKOR technique. Apan et al. (2018) ranked food and beverage sector firms by using the 2008–2014 period. They applied Altman Z-score to determine the financial failures and the VIKOR method was employed to determine the rankings of the financial success of the firms.

Additionally, in this study, tourism companies are ranked according to the two MCDM methods, which are TOPSIS and VIKOR. The MCDM methods are used for ranking in the various parts of tourism. Some of the selected works are as follows. According to Zhang et al. (2011), the TOPSIS ranking method was applied to evaluate the tourism destination competitiveness of the Yangtze River Delta in China, which was weighted by the information entropy weight. Fu et al. (2011) applied the VIKOR ranking method to perform a benchmarking analysis in the hotel industry. Huang and Peng (2012) used the TOPSIS method to evaluate the tourism destination competitiveness of nine Asian countries. Wu et al. (2019), applied the VIKOR method for the evaluation of the rural tourism projects’ financial risk. Nilashi et al. (2019), adopted the TOPSIS method to rank the factors affecting medical tourism in Malaysia. Zheng and Wang (2020) developed an evaluation criterion framework for the renewable energy system, which includes three primary criteria and nine sub-criteria. They ranked the renewable energy system schemes with the VIKOR approach.

## 4 Performance analysis

The structure of the analysis is as follows. In the first step of the analysis, problem definition takes place. In the second step, financial ratios were determined and they were calculated separately for each of the firms. In the third step, the weights of the criteria are first determined by using the entropy method. In the fourth step, TOPSIS and VIKOR methods are used to rank the tourism companies. The entropy method was preferred in determining criterion weights because it eliminates human-induced errors in criteria that can be measured objectively, and is a more realistic objective weighting method. TOPSIS and VIKOR methods are preferred when benefit and cost-oriented criteria are in question. In the last step, a comparison of TOPSIS and VIKOR methods takes place.

### 4.1 Data

This study uses financial ratios to show the financial performances of the tourism companies. In this way, businesses will be able to measure their financial success by considering more than one financial criterion and will have the opportunity to compare them with businesses in the same field of activity, and most importantly with their competitors. Businesses that see that their financial performance is high; will try to keep this situation going. Businesses with lower financial performance compared to others will try to increase their financial performance by making adjustments and improvements in their determined financial criteria. In addition, the fact that the growth rates were not used in the studies conducted for the tourism sector increased the originality of the study.

The data used in the study are the most up-to-date data of the ten tourism companies offered to the public in BIST for the period 2018–2020. The financial information of these companies is taken from Public Disclosure Platform. The data for each of the companies examined within the scope of the study was taken from the audited financial statements. The names of the companies in the study are indicated by their abbreviations in BIST.

### 4.2 Flowchart of the analysis

**Figure 1:**
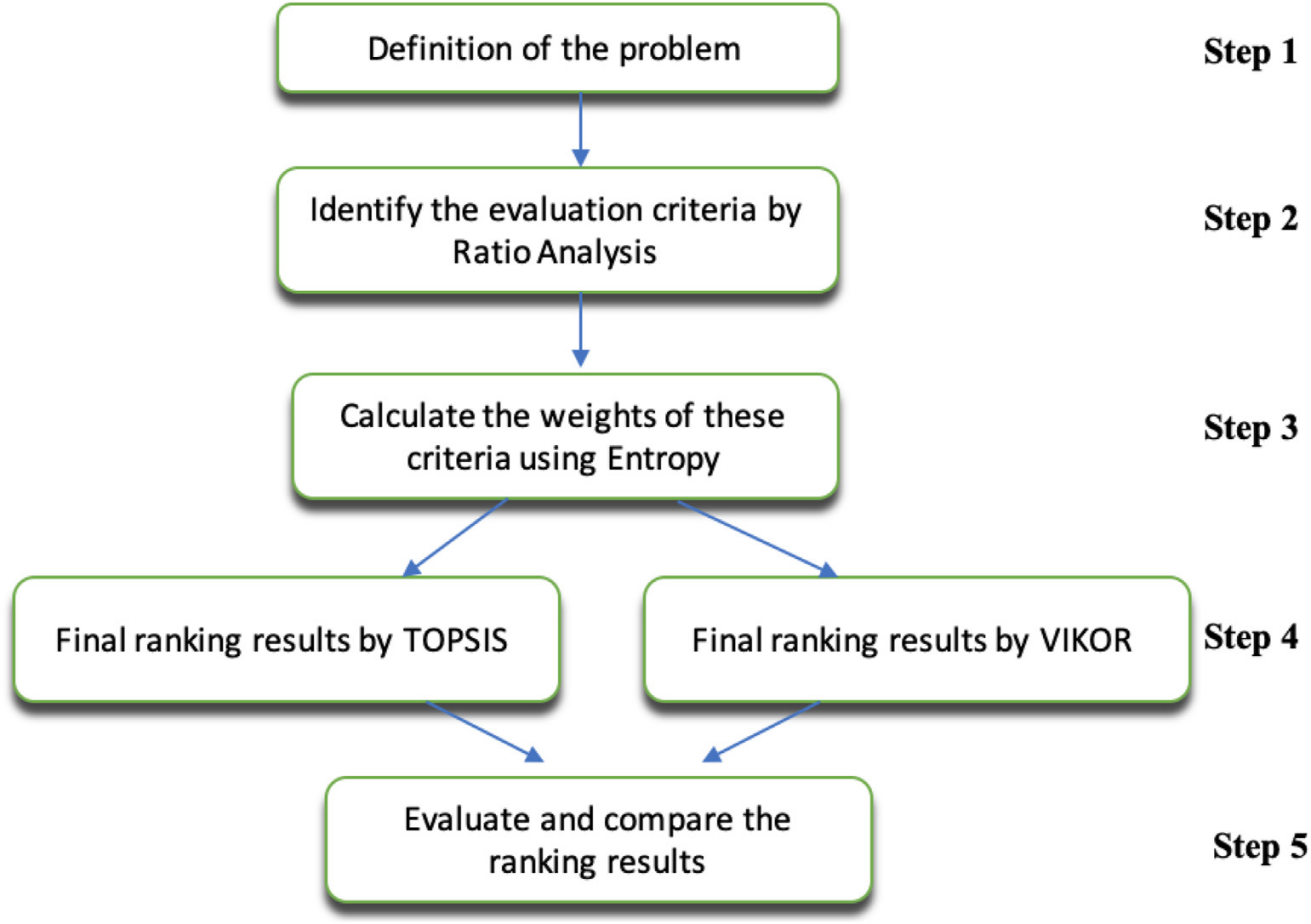
Flow chart of the study.

This paper has five key evaluation steps, as shown in Figure 1. First, the definition of the problem will be stated. Second, the evaluation criteria by ratio analysis will be identified. Third, the weights of these criteria using Entropy will be calculated. Fourth, ranking results by TOPSIS and VIKOR will be presented, and finally, the comparison of the ranking results will take place.

### 4.3 Defining the problem

There are various factors that impact the precision and accuracy of the ultimate answer in a decision-making problem. Each MCDM approach has its own functionality, formulation, and application, which have an impact on the decision-making procedure, the evaluation system path, and the ultimate ranking of alternatives. The combination of the components, including decision variables, normalization instruments, their qualities and weights, and the formulation of the final answer, is what distinguishes these strategies (Wang et al., 2007). As a result, MCDM procedures are an effective tool for ranking or selecting optimal alternatives from a pool of viable options when several, often competing, criteria are available. MCDM approaches were created with the goal of favoring alternatives in a restricted number of categories, with the alternatives ordered in subjective preference order. When many criteria are in conflict, it aids people or systems in making decisions based on their preferences by establishing a goal. The MCDM approach divides complicated decision issues into smaller parts, allocates weight with some care, makes judgments for smaller components, and then reorganizes these smaller components to execute system decision making (Nayak et al., 2020). Thus, the MCDM aims to achieve an optimal decision that is satisfied with all the required characteristics. Decision-making problems need a huge number of factors of varying relevance. MCDM methodologies are frequently effective in guiding the decision-making process to the best solution for a given situation.

Performance evaluation is formulated as a typical MCDM problem, which picks an option from a series of choices described by its features and associated with different parameters for performance value. In this study, entropy-based TOPSIS and VIKOR methods are proposed to deal with financial evaluation problems. The proposed method is applied to a real case; the tourism index of Turkey. Managers have to take many decisions according to their performance. Performance evaluation decisions are conflicting in nature so that managers are keen to know the performance of the other companies and their ranking in the sector. Thus, the problem of the study is to determine the criteria that reveal the performance value and rank the companies according to their values in line with these criteria.

Each MCDM method begins with a decision matrix that includes alternatives and criteria, and the mathematical steps of TOPSIS and VIKOR are presented in Table 1. The steps of the three methods with their formula and detailed explanations are shown in the table below.

In MCDM problems, there are several approaches for determining the weights of criteria. Subjective and objective weighting methods are two types of weighting procedures. Objective approaches are better for avoiding subjectivity issues and decision makers' choices, particularly when the data in the decision matrix is available. Where reliable subjective weights cannot be established, the entropy technique aids in the generation of faster and more accurate criterion weights. The concept of entropy suggests that one of the most important measures of correctness and dependability is the quality of information gained via the decision-making process. In this study, the entropy method has been applied to assign weights of criteria (Odu, 2019). To solve the MCDM problem, we have to follow the 5 steps of the TOPSIS procedure. First, a decision matrix will be created for a multi-criteria decision problem with m alternatives and n criteria. Where: X_ij_: i is alternative, j is the success value according to the criteria, i = 1,2… m and j = 1,2…, n. Second, since the criteria have different scales, the normalization process should be carried out first, and this is done using the following equation. Here; *i* = alternatives, *j* = criteria, r_*ij*_ = normalized values, x_*ij*_ = utility values. Third, entropy values for criteria will be found. Fourth, the degree of differentiation of information will be calculated. Here the value of k is a constant defined by k = 1/lnm and guarantees condition 0 ≤ e_j_≤1. By using the entropy value, the degree of differentiation d_j_, its values are calculated for each criterion as in the formula. Last, the entropy weights of the criteria will be calculated (see Table 1).

Besides, TOPSIS is used to resolve conflicts between criteria that are comparable. The TOPSIS approach was created as a typical multi-criteria decision-making process with to arrive at non-inferior answers. It's simple computational technique and user-friendly structure make a decision problem more dependable, resulting in optimal answers (Nayak et al., 2020). To solve the MCDM problem, we have to follow the 6 steps of the TOPSIS procedure. First, the decision matrix will be created. The decision matrix created by the decision-maker is a matrix of size m x n. i is success values of alternative according to all criteria, column x_jj_ are the success values of all alternatives according to the criteria. Second, the normalization process will be performed. At this step, it is ensured that the matrix is normalized by taking the square root of the sum of squares of the values of the criteria in the decision matrix. An element of the normalized decision matrix is denoted by "*r*_*ij*_". Third, the weighted normalized matrix will be constructed. The previously obtained normalized matrix will be multiplied with the criterion weights matrix that has been previously determined. Fourth, ideal (A ∗) and negative ideal (A⁻) solution points will be created. Here, the maximum and minimum values in each column in the weighted matrix will be determined. Fifth, distances to the maximum ideal point will be calculated. Euclidean metric will be used for distance calculation. Last, relative proximity to the ideal solution will be computed. The relative proximity of each decision point to the ideal solution is calculated and indicated by. The alternatives are sorted by ranking the C_i_∗ values from the highest to the lowest (see Table 1).

Moreover, the VIKOR method is used for financial performance evaluation problems. The VIKOR technique was designed to establish a compromise ranking list of numerous alternatives with non-commensurable and conflicting criteria and specific weights stability intervals so that preference stability and provided weights may be adjusted (Nayak and Tripathy, 2018) VIKOR is introduced as an applied instrument when decision specialists are unable to communicate their choices throughout the system design phase due to its unique structure. Because each option in VIKOR is evaluated using an aggregate function, the compromise ranking of alternatives is accomplished by comparing the measure of proximity to the ideal answer. The VIKOR rating results might be influenced by the omission or addition of an alternative. For the opponent and the majority, the VIKOR method calculates the minimal individual regret and the maximum group utility. To solve the MCDM problems we have to follow the 7 steps of the VIKOR procedure. First, the decision matrix will be created. A decision matrix is created such that rows show alternatives (m) and columns show criteria (n). Second, the best and worst values will be detected. The best (f∗) and worst (f^−^) values are determined for each criterion. If j. If the criterion has the utility property, the parent formula, if j. If the criterion has the cost property, a sub formula is used. Third, a normalized decision matrix will be created. The linear normalization process is applied in this step. Fourth, a weighted decision matrix will be created. The weighted normalized matrix will be obtained by performing the same operations with the 3^rd^ step of the TOPSIS method. Fifth, Sj and Rj values of each alternative will be calculated. Here, *i* denotes mean and worst group scores for an alternative. Sixth, Qj values for each alternative will be calculated. Here, parameter q is the weight of the majority of the criteria, that is, the weight for the strategy that provides the maximum group benefit, and the parameter (1-q) is the weight of the minimum regret. Compromise is achieved by q˃0.5 majority vote, q = 0.5 consensuses, or q˂0.5 veto. Last, alternatives will be ranked (See Table 1).

### 4.4 Performance evaluation

The evaluation of a company's financial performance is of great significance to managers, creditors, and potential investors in today's competitive global economy, as well as for companies in the same sector. Company performance evaluations are usually performed using financial analyses. The notion of financial performance is examined for numerous purposes such as return, productivity, production, and economic growth and may be adapted for both businesses and associated industries through the financial ratios in the performance evaluation process. Financial ratios obtained from income statement and balances sheet data are regarded as significant instruments for measuring organizations' performance and financial assets. A large amount of literature research has demonstrated the advantages of financial ratios for many years. Ratios may be summarized and analyzed by users to offer significant information for decision-making. In terms of liquidity, growth, and profitability, they also show the financial parameters that represent strong and weak sides for businesses.

This study uses financial ratios for financial performance evaluation since financial ratios give a standardized technique for comparing businesses and sectors. In the viewpoint of analysts, using ratios sets all organizations on an equal basis; companies are assessed on their performance instead of their size, the volume of sales, or market share. Evaluating raw financial data from two firms in the same sector provides quite a small amount of information. Ratios reflect a company's ability to make a profit, finance the business, develop via sales instead of debt, and a variety of other aspects beyond the numbers.

In this study, 6 different ratio classes have been created for different goals of different classes for performance evaluation of the companies. These classes can be counted as liquidity, activity, financial structure, profitability, market, and growth to evaluate market performance. The ratios used in this study are given in the table below.

### 4.5 Weighting criteria

Criteria weighting in multi-criteria decision-making methods has a substantial impact on the final result of decision-making and ranking options that engage in the model. Therefore, these weights must be accurately determined. The weights of parameters can be determined using different measures and they can both be divided into two groups as subjective and objective approaches. Subjective approaches are the measures that best value the subjective interests of the decision-maker or specialist in the process of evaluating the validity of parameters. Besides, objective approaches are oriented assigning weight coefficients based on the interpretation of given results, which are then used to solve complex mathematical models of multi-criteria decision-making methods without given decision-makers' or experts' attitudes. Entropy method is considered as a widely used objective method.

This study reviews alternative methods after evaluating objective weight parameters by using algorithms where the relative value of criteria corresponds to the quantity of knowledge found in each criterion and is linked to the contrast strength of each criterion. Moreover, by using the entropy process, the form of criterion becomes unimportant, and it reduces the possibility of subjective criteria weighting errors. In this study entropy method is used for the evaluation of criteria weighting whereas TOPSIS and VIKOR methods are adopted to measure and rank the financial performances of the companies. In addition, objective interface dimension and other supporting aspects have been integrated into the proposed metric to enable the user to obtain the required information during the specified time, which is also essential. Entropy as an analytical approach for determining weights is an appropriate method since it can quantify the homogeneity of individual decisions of both businesses and entrepreneurs. It is used as a method to solve a problem of criteria weighting in the process of financial performance as favorable to business.

In the study, 6 main criteria, namely liquidity, activity, financial structure, profitability, market and growth rates, were determined, besides 3–4 sub-criteria were created for each main criterion, and a total of 20 sub-criteria were created. As the decision maker, the academician's opinion was taken. The relative relevance of the parameters calculated using the entropy technique is displayed in Table 3.

**Table 3:** Weight values.

2018	CR	AR	CAR	AT	CT	ART	IT	LR	DER	LAR	
**wi**	0,013	0,018	0,115	0,019	0,047	0,015	0,029	0,037	0,112	0,027	
	**PBR**	**PSR**	**PER**	**RA**	**RE**	**GPM**	**NPM**	**AGR**	**EGR**	**SGR**	**Total**
**wi**	0,047	0,055	0,015	0,027	0,060	0,047	0,206	0,021	0,050	0,037	1,00
**2019**	**CR**	**AR**	**CAR**	**AT**	**CT**	**ART**	**IT**	**LR**	**DER**	**LAR**	
**wi**	0,017	0,014	0,119	0,015	0,051	0,011	0,033	0,033	0,116	0,023	
	**PBR**	**PSR**	**PER**	**RA**	**RE**	**GPM**	**NPM**	**AGR**	**EGR**	**SGR**	Total
**wi**	0,051	0,051	0,018	0,024	0,064	0,043	0,210	0,017	0,054	0,033	1,00
**2020**	**CR**	**AR**	**CAR**	**AT**	**CT**	**ART**	**IT**	**LR**	**DER**	**LAR**	
**wi**	0,016	0,015	0,118	0,016	0,049	0,013	0,031	0,035	0,114	0,025	
	**PBR**	**PSR**	**PER**	**RA**	**RE**	**GPM**	**NPM**	**AGR**	**EGR**	**SGR**	Total
**wi**	0,049	0,053	0,017	0,025	0,058	0,048	0,207	0,019	0,052	0,037	1,00

According to Table 3, the highest weight among the sub-criteria for 2018, 2019 and 2020 is the net profit margin with the value of 0,206, 0,210 and 0,207 respectively. The second highest weight is the cash ratio with 0,115, 0,119 and 0,118 respectively. The third highest weight is the debt equity ratio with 0,112, 0,116 and 0,114 respectively. Among the sub-criteria, the lowest weight was the current ratio with a value of 0.013 in 2018, accounts receivable turnover with a value of 0,011 in 2019 and 0,013 in 2020.

### 4.6 Ranking of the companies

The weighted criteria have been completed using the entropy method. It has been applied to uncover the comparative importance of the criteria. The step of the ranking alternative has been started with comparative methods by the calculation of the weighted criteria with entropy. The first method for the ranking alternative is the TOPSIS method. Tables 4, 5, and 6 displays the positive and negative ideal measures and Table 7 displays the final ranking according to the TOPSIS method.

**Table 4:** Forming ideal (A ∗) and negative ideal (A-) solution sets.

2018	CR	AR	CAR	AT	CT	ART	IT	LR	DER	LAR
**A∗**	0,005	0,007	0,093	0,012	0,038	0,005	- 0,000	0,024	0,106	0,019
**A-**	0,000	0,000	0,000	0,000	0,000	0,000	- 0,012	0,002	0,001	0,001
	**PBR**	**PSR**	**PER**	**RA**	**RE**	**GPM**	**NPM**	**AGR**	**EGR**	**SGR**
**A∗**	0,031	0,033	0,005	0,009	0,036	0,034	0,164	0,006	0,020	0,014
**A-**	0,001	0,001	0,000	0,001	0,000	0,001	0,000	0,000	0,000	0,000
**2019**	**CR**	**AR**	**CAR**	**AT**	**CT**	**ART**	**IT**	**LR**	**DER**	**LAR**
**A∗**	0,004	0,008	0,089	0,016	0,037	0,006	- 0,000	0,021	0,109	0,017
**A-**	0,000	0,000	0,000	0,000	0,000	0,000	- 0,011	0,002	0,001	0,001
	**PBR**	**PSR**	**PER**	**RA**	**RE**	**GPM**	**NPM**	**AGR**	**EGR**	**SGR**
**A∗**	0,030	0,035	0,004	0,010	0,035	0,039	0,159	0,008	0,018	0,016
**A-**	0,001	0,001	0,000	0,001	0,000	0,001	0,000	0,000	0,000	0,000
**2020**	**CR**	**AR**	**CAR**	**AT**	**CT**	**ART**	**IT**	**LR**	**DER**	**LAR**
**A∗**	0,003	0,009	0,089	0,016	0,037	0,006	- 0,000	0,027	0,103	0,016
**A-**	0,000	0,000	0,000	0,000	0,000	0,000	- 0,013	0,001	0,001	0,001
	**PBR**	**PSR**	**PER**	**RA**	**RE**	**GPM**	**NPM**	**AGR**	**EGR**	**SGR**
**A∗**	0,029	0,034	0,006	0,008	0,038	0,032	0,162	0,005	0,021	0,017
**A-**	0,001	0,001	0,000	0,001	0,000	0,001	0,000	0,000	0,000	0,000

**Table 5:** Positive ideal (S ∗) discrimination measures.

2018	TEKTU	AYCES	AVTUR	MARTI	UTPYA	MERIT	KSTUR	PKENT	MAALT	ULAS
**S∗**	0,209	0,236	0,139	0,239	0,230	0,231	0,232	0,233	0,165	0,220
**2019**	**TEKTU**	**AYCES**	**AVTUR**	**MARTI**	**UTPYA**	**MERIT**	**KSTUR**	**PKENT**	**MAALT**	**ULAS**
**S∗**	0,210	0,237	0,140	0,241	0,231	0,233	0,235	0,236	0,166	0,221
**2020**	**TEKTU**	**AYCES**	**AVTUR**	**MARTI**	**UTPYA**	**MERIT**	**KSTUR**	**PKENT**	**MAALT**	**ULAS**
**S∗**	0,206	0,233	0,136	0,236	0,227	0,228	0,229	0,230	0,162	0,217

**Table 6:** Negative ideal (S ∗) discrimination measures.

2018	TEKTU	AYCES	AVTUR	MARTI	UTPYA	MERIT	KSTUR	PKENT	MAALT	ULAS
**S∗**	0,120	0,136	0,186	0,047	0,053	0,045	0,047	0,048	0,109	0,098
**2019**	**TEKTU**	**AYCES**	**AVTUR**	**MARTI**	**UTPYA**	**MERIT**	**KSTUR**	**PKENT**	**MAALT**	**ULAS**
**S∗**	0,121	0,138	0,187	0,048	0,055	0,046	0,048	0,049	0,110	0,099
**2020**	**TEKTU**	**AYCES**	**AVTUR**	**MARTI**	**UTPYA**	**MERIT**	**KSTUR**	**PKENT**	**MAALT**	**ULAS**
**S∗**	0,118	0,133	0,184	0,045	0,051	0,042	0,045	0,046	0,106	0,095

**Table 7:** Calculation of proximity according to the ideal solution.

2018	TEKTU	AYCES	AVTUR	MARTI	UTPYA	MERIT	KSTUR	PKENT	MAALT	ULAS
**C+**	0,365	0,166	0,571	0,136	0,189	0,169	0,170	0,171	0,397	0,305
**Ranking**	3	9	1	10	5	8	7	6	2	4
**2019**	TEKTU	AYCES	AVTUR	MARTI	UTPYA	MERIT	KSTUR	PKENT	MAALT	ULAS
**C+**	0,389	0,169	0,528	0,159	0,194	0,165	0,181	0,186	0,472	0,341
**Ranking**	3	8	1	10	5	9	7	6	2	4
**2020**	TEKTU	AYCES	AVTUR	MARTI	UTPYA	MERIT	KSTUR	PKENT	MAALT	ULAS
**C+**	0,357	0,153	0,472	0,131	0,175	0,161	0,168	0,164	0,489	0,296
**Ranking**	3	9	2	10	5	8	6	7	1	4

The maximum values in each column for the positive ideal solution and the minimum values in each column for the negative ideal solution were taken into account. Positive and negative ideal solution sets were formed by using equations 9 and 10.

The positive ideal discrimination criterion was formed by using equation 11.

The negative ideal discrimination criterion was established using equation 12.

Proximity calculation was made according to the ideal solution using equation 13 and the companies were ranked. As a result of the ranking made with TOPSIS method; it was determined that AVTUR firm had the best performance in 2018, and MARTI had the worst performance. It was determined that in 2019, AVTUR had the best performance and MARTI had the worst performance. In 2020, MAALT showed the best performance, and it was determined that MARTI had the worst performance.

The second method used in this study for a comparative analysis is the VIKOR method. Tables 8, 9, and 10 displays the S, R, Q values and final ranking list according to the VIKOR method.

**Table 8:** Calculation of Sj and Rj values of each alternative.

2018	TEKTU	AYCES	AVTUR	MARTI	UTPYA	MERIT	KSTUR	PKENT	MAALT	ULAS
**Si**	0,626	0,779	0,600	0,801	0,787	0,778	0,776	0,764	0,662	0,688
**Ranking**	2	8	1	10	9	7	6	5	3	4
**Ri**	0,208	0,207	0,108	0,206	0,193	0,205	0,204	0,203	0,106	0,208
**Ranking**	10	8	2	7	3	6	5	4	1	9
**2019**	TEKTU	AYCES	AVTUR	MARTI	UTPYA	MERIT	KSTUR	PKENT	MAALT	ULAS
**Si**	0,648	0,761	0,617	0,768	0,742	0,711	0,735	0,704	0,626	0,633
**Ranking**	4	9	1	10	8	6	7	5	2	3
**Ri**	0,202	0,132	0,109	0,195	0,200	0,179	0,181	0,145	0,104	0,209
**Ranking**	9	3	2	7	8	5	6	4	1	10
**2020**	TEKTU	AYCES	AVTUR	MARTI	UTPYA	MERIT	KSTUR	PKENT	MAALT	ULAS
**Si**	0,599	0,743	0,578	0,800	0,793	0,777	0,687	0,712	0,632	0,655
**Ranking**	2	7	1	10	9	8	5	6	3	4
**Ri**	0,209	0,204	0,106	0,205	0,168	0,207	0,181	0,202	0,125	0,208
**Ranking**	10	6	1	7	3	8	4	5	2	9

**Table 9:** Calculating Qj values for each alternative.

2018	TEKTU	AYCES	AVTUR	MARTI	UTPYA	MERIT	KSTUR	PKENT	MAALT	ULAS
**V = 0,25**	0,779	0,896	0,009	0,919	0,824	0,893	0,892	0,890	0,059	0,830
**Ranking**	3	9	1	10	4	8	7	6	2	5
**V = 0,50**	0,559	0,797	0,006	0,843	0,758	0,792	0,789	0,787	0,120	0,663
**Ranking**	3	9	1	10	5	8	7	6	2	4
**V = 0,75**	0,340	0,697	0,002	0,871	0,691	0,780	0,771	0,767	0,181	0,496
**Ranking**	3	6	1	10	5	9	8	7	2	4
**V = 1**	0,120	0,597	0	0,690	0,625	0,783	0,794	0,831	0,241	0,329
**Ranking**	2	5	1	7	6	8	9	10	3	4
**2019**	TEKTU	AYCES	AVTUR	MARTI	UTPYA	MERIT	KSTUR	PKENT	MAALT	ULAS
**V = 0,25**	0,796	0,713	0,009	0,904	0,898	0,865	0,891	0,833	0,643	0,082
**Ranking**	5	4	1	10	9	7	8	6	3	2
**V = 0,50**	0,508	0,631	0,005	0,831	0,809	0,729	0,768	0,626	0,431	0,123
**Ranking**	4	5	1	10	9	7	8	6	3	2
**V = 0,75**	0,357	0,456	0,002	0,816	0,647	0,724	0,765	0,667	0,179	0,167
**Ranking**	4	5	1	10	6	8	9	7	3	2
**V = 1**	0,369	0,591	0	0,611	0,675	0,746	0,699	0,801	0,120	0,223
**Ranking**	4	6	1	7	5	9	8	10	2	3
**2020**	TEKTU	AYCES	AVTUR	MARTI	UTPYA	MERIT	KSTUR	PKENT	MAALT	ULAS
**V = 0,25**	0,148	0,859	0,008	0,871	0,496	0,869	0,731	0,827	0,049	0,639
**Ranking**	3	8	1	10	4	9	6	7	2	5
**V = 0,50**	0,345	0,762	0,007	0,823	0,628	0,783	0,719	0,747	0,126	0,543
**Ranking**	3	8	1	10	5	9	6	7	2	4
**V = 0,75**	0,326	0,791	0,003	0,868	0,561	0,680	0,778	0,788	0,184	0,435
**Ranking**	3	9	1	10	5	6	7	8	2	4
**V = 1**	0,117	0,721	0	0,699	0,685	0,623	0,786	0,768	0,241	0,318
**Ranking**	2	8	1	7	6	5	10	9	3	4

**Table 10:** Calculating Qj values for each alternative.

2018					
**1**	**DQ**	0,423	**Q″-Q′**	0,487	**V = 0,25**
**1**	**DQ**	0,423	**Q″-Q′**	0,103	**V = 0,50**
**1**	**DQ**	0,423	**Q″-Q′**	0,167	**V = 0,75**
**1**	**DQ**	0,423	**Q″-Q′**	0,110	**V = 1,00**
**2019**					
**1**	**DQ**	0,424	**Q″-Q′**	0,496	**V = 0,25**
**1**	**DQ**	0,424	**Q″-Q′**	0,107	**V = 0,50**
**1**	**DQ**	0,424	**Q″-Q′**	0,178	**V = 0,75**
**1**	**DQ**	0,424	**Q″-Q′**	0,115	**V = 1,00**
**2020**					
**1**	**DQ**	0,421	**Q″-Q′**	0,465	**V = 0,25**
**1**	**DQ**	0,421	**Q″-Q′**	0,101	**V = 0,50**
**1**	**DQ**	0,421	**Q″-Q′**	0,156	**V = 0,75**
**1**	**DQ**	0,421	**Q″-Q′**	0,108	**V = 1,00**

S and R values were calculated by using equations 21 and 22.

Q values were calculated using equation 23.

The conditions were checked using equation 24.

As a result of the ranking made with VIKOR method; it was determined that AVTUR firm had the best performance in 2018, and MARTI had the worst performance. In 2019, it was determined that AVTUR had the best performance and MARTI had the worst performance. In 2020, it was determined that AVTUR had the best performance and MARTI had the worst performance.

## 5 Results and discussion

The financial tables are used to evaluate the performance of businesses in this research, which employs a common multi-criteria decision-making procedure. The suggested technique compares enterprises in the same industry to calculate their ranking based on the criteria established for each year. The comparison of each year's ranking results allows us to identify tourism companies with consistent financial outcomes. It assists businesses in revising their financial data and analyzing their financial status. This study uses the market and growth ratios different than other studies. The market ratios provide information on a firm's position in the market. As a result, it is frequently utilized for financial analysis and company comparison. The growth ratios are a gauge of a company's performance as well as an indication of how the market perceives the company's future growth possibilities.

Each criterion is assumed to have a scale factor in both TOPSIS and VIKOR approaches. This scale demands that all parameters values be eliminated in their different units. An aggregating function is used to rank the values that have been determined by methods. The aggregation techniques are the key distinction between the two processes. The VIKOR approach uses an aggregating function to describe the distances between ideal and non-ideal solutions, and offers a consensus solution with a rate of advantage, in addition to TOPSIS. Each approach has its own set of normalization procedures. The VIKOR method employs linear normalization, whereas the TOPSIS method employs vector normalization. The normalized value of linear normalization is unaffected by the criteria's unit. In the TOPSIS process, the normalized value for a given criterion can vary depending on the evaluation unit.

The TOPSIS approach proposes a ranking index that takes into account the distances between the ideal and negative-ideal points. TOPSIS adds all distances together without taking into account their relative value. The TOPSIS approach employs n-dimensional Euclidean distance, which may represent a balance between overall and individual satisfaction on its own, but does so in a different way than VIKOR, which employs weight v. A ranking list is generated by both approaches. According to the VIKOR approach, the highest-ranked alternative is the closest to the ideal solution. Conversely, according to the TOPSIS approach, the highest ranked alternative does not have to be the closest to the ideal solution.

**Chart 1:**
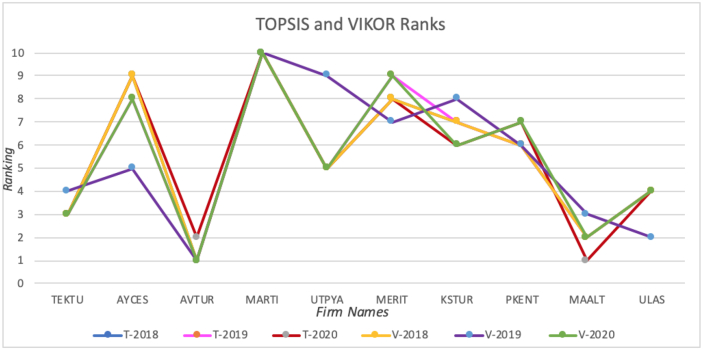
TOPSIS and VIKOR ranks.

**Chart 2:**
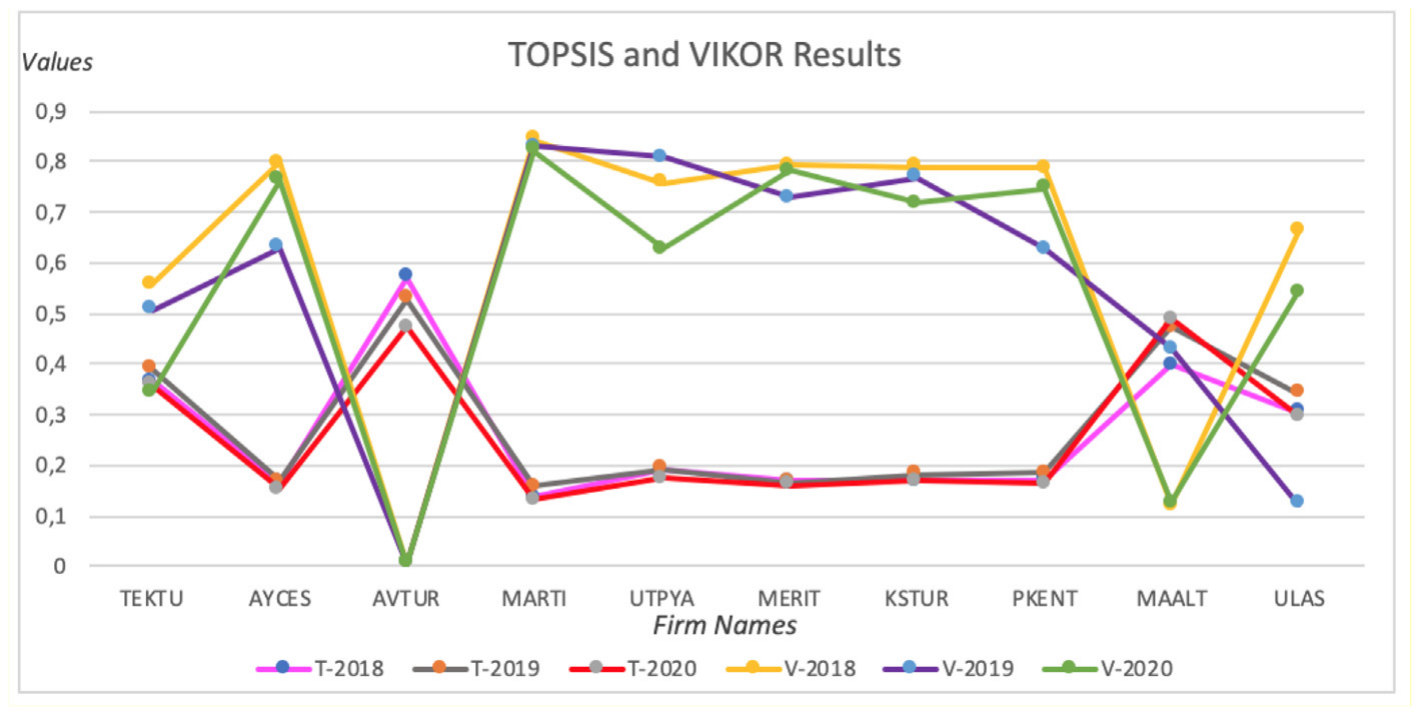
TOPSIS and VIKOR results.

Chart 1 presents the TOPSIS and VIKOR rankings of the alternatives for three years. Chart 2 shows the results of TOPSIS using linear normalization and absolute values, and VIKOR using absolute values.

In the analysis results regarding the evaluation of the financial performance of tourism companies traded in BIST, it was seen that the ranking results made with TOPSIS and VIKOR methods were similar in 2018 and 2019. It is slightly different in 2020. It was seen that AVTUR was the most important alternative in both methods, whereas MARTI had the lowest ranking alternative. Moreover, MERIT, KSTUR, and PKENT have been determined as fluctuating companies.

## 6 Conclusion

Investors that trade in the competitive capital markets are eager to face accountability for their own investment outcomes. Most stock market traders are likely to make poor decisions at the wrong moment. Traders at the stock market do not take the trends often and respond late as a crowd-follower. Therefore, the MCDM analysis approach for stock selection gives the knowledge and investment instruments required at the proper moment. However, stock traders must choose the right indicators and read results correctly.

This paper offers a comparative empirical analysis on the MCDM, which examines the stock performance outcomes with entropy-based TOPSIS and VIKOR techniques in tourism companies. In the study, performance evaluation was done by ratio analysis with 6 main ratio classes in a total of 20 ratios. Financial ratios give both investors and analysts helpful quantitative financial information to evaluate their operations and to study their position over time within a sector. In this regard, this study presents a model suggestion for the financial performance evaluation of the 10 tourism companies that use financial ratios to assess their efficient and productive performance.

The entropy method was employed to determine the criteria weight. TOPSIS and VIKOR methods were used for ranking the results of the best performing and worst-performing companies. Through the methods used in the study, the financial performances of the firms were evaluated, and an evaluation system in which their rankings among themselves were expressed mathematically was introduced. It has been observed that the results obtained as a result of these methods support each other.

TOPSIS and VIKOR methods, which are among the multi-criteria decision-making methods, were used in the (10 × 20) dimensioned Standard Decision Matrices organized separately for each year in the 2018–2020 period. It has been converted into a single score that shows the financial performance of the tourism firms traded on the BIST. However, two systems with distinct procedures for sorting the alternative have the same performance outcomes when it comes to ranking the tourism companies, particularly for the top lines. Overall, the methods' performance reveals that both techniques produce consistent results when ranking tourism companies.

In the study, it has been observed that the performances of the enterprises with high market rates are high and if they are further reduced, the price/sales ratio of these enterprises is higher than the market rates. It may be suggested that companies to be analyzed with entropy-based TOPSIS and VIKOR methods should focus on market rates and also on the price/sales ratio among these. Businesses know how much they will pay for their sales in each unit and can estimate the value of their future investments. This will show that companies can act consistently in terms of price in their purchases and sales. The high price/sales ratio may affect the conditions of the companies positively. In addition, it may be suggested that multi-criteria decision-making methods should be evaluated in other sectors in future studies. The most important constraint of this study is that the results may change as the use of financial ratios used in the study changes.

For further research, the assessment approach may be integrated with other ranking techniques and industries for future investigation. Furthermore, the study's criteria may be expanded with the key patterns of the MCDM analysis, which are suited for comparative assessment. Decision-makers from various sectors, together with capital market professionals, might be tasked with setting the criteria and rating the different sectors based on the findings of the indicators and patterns. The predicted outcome may then be used as a confirmatory determinant to assess the overall performance of all sectors and the capital market.

## Declarations

### Author contribution statement

Nida Türegün: Conceived and designed the experiments; Performed the experiments; Analyzed and interpreted the data; Contributed reagents, materials, analysis tools or data; Wrote the paper.

### Funding statement

This research did not receive any specific grant from funding agencies in the public, commercial, or not-for-profit sectors.

### Data availability statement

The author does not share their data but the public data can be accessed through Borsa Istanbul website.

### Declaration of interests statement

The authors declare no conflict of interest.

### Additional information

No additional information is available for this paper.
